# The association between blood urea nitrogen to albumin ratio and cognitive function in Parkinson’s disease patients

**DOI:** 10.3389/fneur.2025.1614862

**Published:** 2026-01-09

**Authors:** Wen Zhou, Qingqing Xia, Duan Liu, Tian-fang Zeng, Rui-juan Pang, Jun-ying Li, Liang Gong

**Affiliations:** Chengdu Second People’s Hospital, Chengdu, Sichuan, China

**Keywords:** biomarker, blood urea nitrogen to albumin ratio, cognitive impairment, Montreal cognitive assessment (MoCA) scale, Parkinson’s disease

## Abstract

**Background:**

Cognitive impairment is a significant complication in Parkinson’s disease (PD), impacting quality of life and increasing caregiver burden. This study investigates the association between the blood urea nitrogen to albumin ratio (BAR) and cognitive impairment in PD patients, aiming to identify BAR as a potential biomarker for early detection and monitoring of cognitive decline.

**Methods:**

Data from 1,312 PD patients were extracted from the PPMI database. The cognitive assessment tool was the Montreal Cognitive Assessment (MoCA) score and other cognitive metrics. The association between BAR and cognitive impairment was assessed using multivariate linear regression models to evaluate the continuous relationship, and logistic regression models to examine the binary outcomes. Furthermore, subgroup and sensitivity analyses were carried out to ensure the reliability of the results.

**Results:**

Higher BAR levels were significantly associated with lower MoCA scores in PD patients, independent of other confounding variables (*β* = −0.21, 95% CI = -0.35 ~ −0.07, *p* = 0.003). The odds ratio for cognitive impairment, defined by a MoCA score cutoff of 26, was 1.15 (95% CI: 1.02–1.30, *p* = 0.027). Further analysis revealed that BAR was negatively correlated with the Benton Judgment of Line Orientation MOANS Scale Score and the Symbol-Digit Modalities Test *T*-score, and positively correlated with the Trail Making Test Part A Reverse *Z*-score. These findings suggest that higher BAR levels are associated with poorer performance in visuospatial abilities, processing speed, and motor function.

**Conclusion:**

This study underscores the potential utility of BAR as a biomarker for cognitive impairment in PD. Future prospective cohort studies are warranted to validate these findings and explore the potential of BAR in clinical practice.

## 1 Introduction

Parkinson’s disease (PD), the second most prevalent neurodegenerative disorder worldwide, currently affects over 1% of individuals aged 65 years and older (1), with a projected doubling of prevalence by 2030 (2). The disease spectrum encompasses both motor and non-motor manifestations, with approximately 2% of patients presenting initial non-motor features (non-motor dominant PD) (3). Neurodegenerative non-motor complications span sensory disturbances, sleep disorders, autonomic dysfunction, and neuropsychiatric impairments, including psychosis, depression, and cognitive decline (3, 4).

Cognitive dysfunction, manifesting as deficits in executive function, visuospatial processing, verbal fluency, processing speed, and complex attention, may emerge at any disease stage, with memory impairment developing in later phases (5, 6). PD-associated dementia occurs at an annual incidence of 10% (7), significantly impairing quality of life, increasing caregiver burden, and reducing survival (7, 8). While pathophysiological mechanisms involve dopaminergic circuit disruption in the basal ganglia and cholinergic transmission deficits in frontal networks, multifactorial contributors include *α*-synuclein aggregation, amyloid-*β* deposition, oxidative stress, neuroinflammation, and mitochondrial dysfunction (9, 10). Identified risk factors for PD dementia include male sex, hypertension, diabetes, hyperuricemia (11), advanced age, longer disease duration, rigidity-bradykinesia phenotype, severe cognitive impairment, semantic fluency deficits, genetic factors, lower education level, and postural instability (5). Current pharmacotherapies demonstrate limited efficacy without disease modification, underscoring the urgent need for novel therapeutic strategies and biomarkers.

The blood urea nitrogen-to-albumin ratio (BAR), a composite marker integrating renal function, nutritional status, and inflammatory responses, has gained attention in research on acute and emergency conditions (12–15). Among patients in the acute and subacute phases of various diseases undergoing rehabilitation, elevated BUN (blood urea nitrogen) levels have been identified as an independent risk factor for delirium (16). However, given the diverse characteristics of high BUN and low albumin levels, BAR is also believed to play a role in the development of chronic diseases. Previous study has found that low serum albumin levels and elevated serum urea nitrogen levels are risk factors for cognitive frailty in elderly patients undergoing maintenance hemodialysis (17). Recent investigations reveal BAR’s association with cerebral small vessel disease progression (18), yet its role in PD-associated cognitive decline remains unexplored.

This study aims to examine the correlation between BAR and cognitive impairment in PD patients, seeking to identify BAR as a simple biomarker for early detection and monitoring of cognitive decline, thereby informing the development of more effective treatment strategies.

## 2 Materials and methods

### 2.1 Participant selection and study grouping

**Figure 1:**
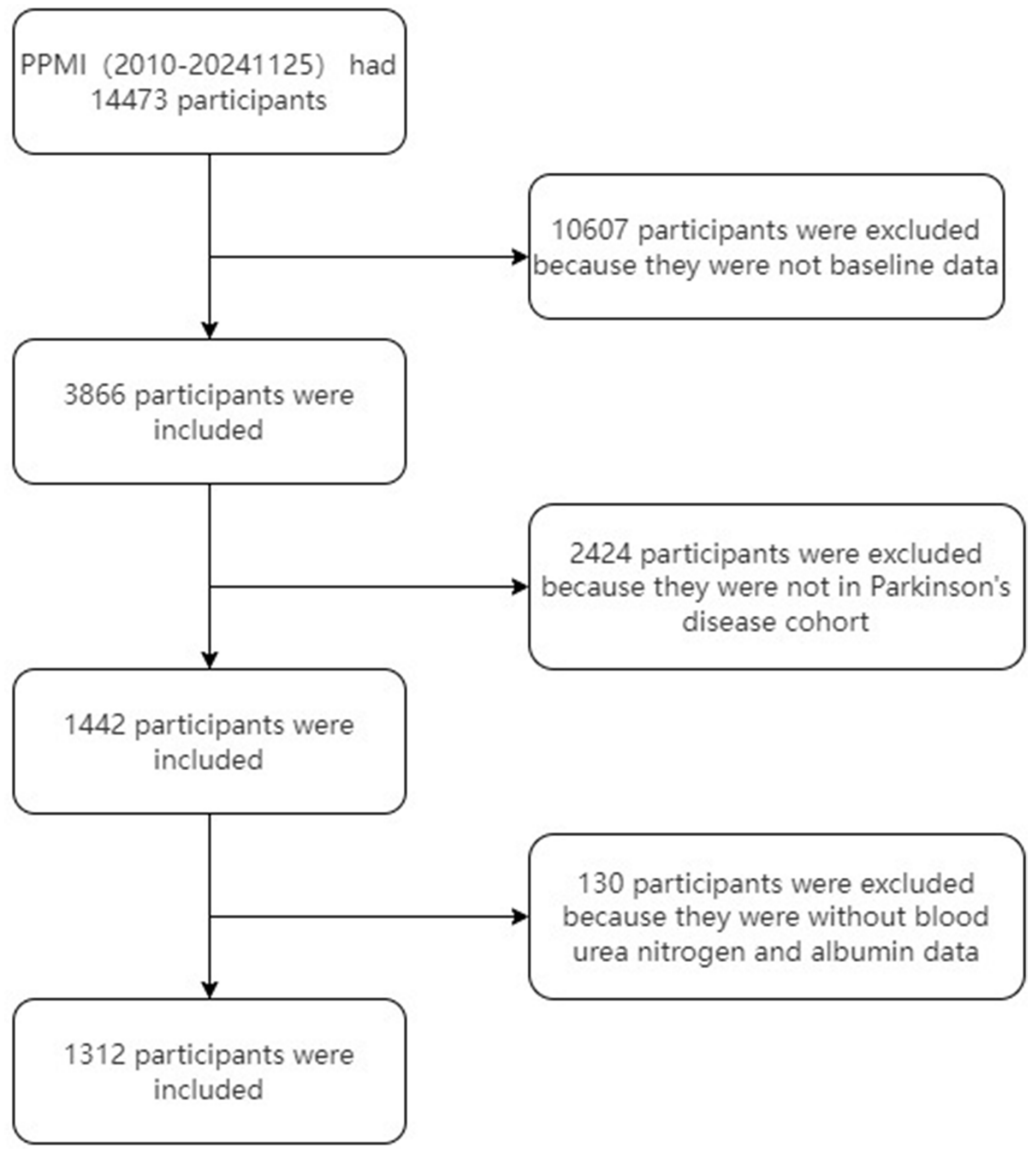
Flowchart of the study cohort.

Data were extracted from the Parkinson’s Progression Markers Initiative (PPMI) database^1^, RRID: SCR_006431, on November 25, 2024. Notably, the study is registered with ClinicalTrials.gov (NCT01141023). The flowchart for participant enrollment is presented in Figure 1. The studies comply with regulations and ethical guidelines, with written informed consent obtained from all participants. The protocol is approved by local Institutional Review Boards, and research activities adhere to the principles of the 1964 Declaration of Helsinki and its revisions.

### 2.2 Inclusion and exclusion criteria for PD patients (adapted from PPMI database)

#### 2.2.1 Inclusion criteria

1. Aged 30 years or older at screening.
2. PD diagnosis within 2 years at screening.
3. No PD medication required within 6 months from baseline.
4. Presence of at least two motor features: resting tremor, bradykinesia, rigidity (with resting tremor or bradykinesia required), or asymmetric resting tremor/bradykinesia.
5. Hoehn and Yahr stage I or II at baseline.
6. Willingness and medical ability to discontinue certain medications (e.g., alpha-methyldopa, methylphenidate) for at least 5 half-lives before SPECT imaging.
7. Eligibility confirmed by screening SPECT imaging.
8. Ability to provide informed consent.
9. For females of childbearing potential: negative pregnancy test on the day of screening SPECT imaging, with no plans for pregnancy or lactation during the study.

#### 2.2.2 Exclusion criteria

1. Current or recent use of PD medications (e.g., levodopa, dopamine agonists) except low-dose treatment for restless leg syndrome.
2. History of atypical PD syndromes (e.g., due to drugs or metabolic disorders).
3. Clinical diagnosis of dementia.
4. Significant neurological disorders on MRI.
5. Recent use of dopamine receptor blockers or other contraindicated drugs.
6. Conditions precluding safe lumbar puncture (e.g., lumbar spinal disease, coagulopathy).
7. Any other medical or psychiatric condition or lab abnormality, which in the opinion of the investigator might preclude participation.
8. Investigator’s discretion regarding suitability for study enrollment.

In the PPMI study, blood samples were collected from PD patients during the baseline visit. Participants were required to fast overnight, and venous blood samples were drawn the next morning. All biochemical analyses were conducted uniformly at Covance laboratories, in accordance with the study protocol. A central laboratory was utilized to ensure identical analysis methods and consistent normal ranges, facilitating a common interpretation of laboratory changes. The biochemical analyses included measurements of alanine aminotransferase (ALT), serum glucose, creatinine, serum uric acid, urea nitrogen, and albumin. These analyses were performed using standardized laboratory techniques to ensure accuracy and reliability. Venous whole blood samples were collected, and all samples for laboratory analysis were collected, prepared, labeled, and transported according to the requirements outlined in the PPMI Laboratory Manual. During screening or baseline visits, a maximum of 60 milliliters of blood was drawn. Specifically, approximately 10 milliliters of whole blood, 30 milliliters of serum, and 10 milliliters of plasma were collected for metabolomics, genetics, and other research analyses. Blood samples were collected in a fasting state, defined as at least 8 h after the last meal or food intake, to ensure sample quality for future analyses. If fasting was not possible, participants were advised to consume a low-fat diet. It is important to note that participants did not receive any individual results from the research analyses or biosample testing. For further detailed information on PPMI biospecimens, the PPMI Biospecimen Manual can be consulted. This approach ensures that the assessment of biochemical markers and other variables is conducted with high precision and consistency, providing reliable data for the study.

In this study, we utilized the Montreal Cognitive Assessment (MoCA) (education-corrected) as our primary tool to evaluate overall cognitive function, given its sensitivity in detecting cognitive impairment in PD. To further explore the relationship between the BAR and cognitive function, we also analyzed a comprehensive set of cognitive metrics from the PPMI database. These included the Clock Drawing Test *T*-score (age-corrected), Hopkins Verbal Learning Test-Revised (HVLT-R) Immediate/Total Recall *T*-score, HVLT-R Delayed Recall *T*-score, HVLT-R Retention *T*-score, HVLT-R Discrimination Index *T*-score, Semantic Fluency (Animal) *T*-score (age and education-corrected), Letter-Number Sequencing *T*-score (age-corrected), Benton Judgment of Line Orientation MOANS Scale Score (age and education-corrected), Boston Naming Test Scale Score (age and education-corrected), Symbol-Digit Modalities Test *T*-score (age and education-corrected), Letter Fluency Test (FAS) *T*-score (age and education-corrected), Trail Making Test Part A Reverse *Z*-score (age and education-corrected), and Trail Making Test Part B Reverse *Z*-score (age and education-corrected). These measures collectively assess various cognitive domains, including visuospatial abilities, verbal learning and memory, working memory, attention, processing speed, and executive function.

### 2.3 Measurement

The BAR was calculated as the ratio of Blood Urea Nitrogen (BUN) (mg/dL) to Albumin (g/dL). This ratio serves as an indicator of renal function and overall metabolic health. The formula used for calculating BAR is:


BAR=BUN(mg/dL)/Albumin(g/dL)

### 2.4 Statistical methods

We assessed the normality of variable distributions using histogram analysis, Q-Q plots, and the Kolmogorov–Smirnov test. Normally distributed continuous variables are presented as mean ± standard deviation (SD), while skewed variables are shown as median (interquartile range [IQR]). Categorical data are expressed as frequencies with percentages.

Statistical comparisons across BAR groups were performed using chi-square or Fisher’s exact tests for categorical variables, one-way ANOVA for normally distributed variables, and the Kruskal-Wallis *H* test for skewed variables.

We used multiple imputation with 5 replications and a chained equation approach in the R mice package to maximize statistical power and minimize bias from missing data (19).

To evaluate the impact of BAR on cognitive impairment in PD patients, we employed linear regression models to assess the relationship between BAR and MoCA scores, presenting results as *β* with 95% confidence intervals (CI). BAR was categorized into tertiles. We constructed three models: Model 1 was unadjusted; Model 2 adjusted for age, sex, race, and BMI; Model 3 further adjusted for ALT, creatinine, serum uric acid, and serum glucose. Covariates were selected based on clinical relevance, literature review, and univariate analysis significance (20–24). Additionally, we categorized MoCA scores into cognitive impairment and non-cognitive impairment groups and used logistic regression to examine the relationship between BAR and cognitive impairment. We also conducted linear regression analyses to explore the relationship between BAR and other cognitive assessment scales.

We converted BAR into a categorical variable by tertile and calculated the P for trend to verify the results of BAR as a continuous variable and to examine non-linearity. Restricted cubic spline (RCS) models with four knots (5th, 35th, 65th, and 95th percentiles) were used to examine potential non-linear dose–response relationships (25). Non-linearity was assessed using a likelihood ratio test comparing models with and without cubic spline terms. We further developed a two-piecewise linear regression model to identify threshold effects, adjusting for potential confounders.

To ensure robustness, we conducted sensitivity analyses by excluding patients with missing covariates. Additionally, we also excluded patients with renal insufficiency to account for potential biases that could arise from compromised kidney function affecting the BUN levels. We also performed subgroup analyses based on age, sex, BMI, PD duration, and PD genetic subtypes to assess the stability of our results.

We performed a longitudinal analysis using all follow-up cognitive assessments available in PPMI. Of 1,312 early-stage PD patients, 6,323 person-visits were retained after excluding visits with missing BAR. Group-based trajectory modelling (GBTM) was applied to repeated 0–30-point MoCA scores using the R package lcmm (v2.0.0). We fitted censored-normal mixture models with linear, quadratic and cubic polynomial terms and compared 2- to 5-group solutions. Optimal number of trajectories was selected by the lowest Bayesian Information Criterion (BIC), combined with (i) average posterior probability ≥0.70, (ii) group size ≥5% of the sample, and (iii) clinical interpretability. A three-group model (entropy = 0.58) provided the best fit and was labeled according to their visual pattern as Stable-MoCA (*n* = 607), Slow-progressing MoCA (*n* = 574) and Fast-declining MoCA (*n* = 131). Each participant was assigned to the trajectory with the highest posterior probability. Multinomial logistic regression was used to relate baseline BAR (per 1-SD increase) to trajectory membership, with Stable-MoCA as the reference category. Three nested models were fitted: Model 1 (crude); Model 2 adjusted for age, sex, race, BMI; Model 3 additionally adjusted for serum glucose, creatinine, ALT, serum uric acid.

All analyses were performed using R Statistical Software (Version 4.2.2^2^, The R Foundation) and Free Statistics analysis platform (Version 1.9, Beijing, China^3^). A two-sided *p* value < 0.05 was considered statistically significant.

## 3 Results

### 3.1 Population characterization

We included 1,312 patients aged 62.9 ± 9.7 years, white was 94.6%, and male were 62.3%. The overall mean score of MoCA was 26.8 ± 2.7. The baseline characteristics of the groups stratified by BAR are shown in Table 1. The three groups differed in age, sex, BMI, PD genetic type, creatinine, serum uric acid, urea nitrogen, albumin, MoCA score, MoCA Cognitive status (Cutoff 26), HVLT-R Retention T-score, HVLT-R Discrimination Index *T*-score, and Symbol-Digit Modalities Test *T*-score (all *p* value < 0.05). Otherwise, the distribution of patients’ characteristics (education years, race, PD duration years, serum glucose, ALT, Clock Drawing Test *T*-score, HHVLT-R Immediate/Total Recall *T*-score, HVLT-R Delayed Recall *T*-score, Semantic Fluency (Animal) *T*-score, Letter-Number Sequencing *T*-score, Benton Judgment of Line Orientation MOANS Scale Score, Boston Naming Test Scale Score, FAS *T*-score, Trail Making Test Part A Reverse *Z*-score, and Trail Making Test Part B Reverse *Z*-score) between BAR groups was similar (all *p* value > 0.05).

**Table 1:** Clinical characteristics of the study population by BAR.

Variables	Total (*n* = 1,312)	BAR			*p*-value
		T1 (*n* = 437)	T2 (*n* = 435)	T3 (*n* = 440)	
Age(year), Mean ± SD	62.9 ± 9.7	59.9 ± 9.8	63.0 ± 9.8	65.9 ± 8.5	<0.001
Sex, *n* (%)					<0.001
Female	494 (37.7)	202 (46.2)	152 (34.9)	140 (31.8)	
Male	818 (62.3)	235 (53.8)	283 (65.1)	300 (68.2)	
Education years (year), Mean ± SD	15.8 ± 3.1	15.7 ± 2.9	15.9 ± 3.0	15.7 ± 3.3	0.412
Race, *n* (%)					0.531
White	1,236 (94.6)	408 (94)	407 (94)	421 (95.9)	
Black	13 (1.0)	5 (1.2)	5 (1.2)	3 (0.7)	
Asian	17 (1.3)	8 (1.8)	7 (1.6)	2 (0.5)	
Other (includes multi-racial)	40 (3.1)	13 (3)	14 (3.2)	13 (3)	
BMI (kg/m^2^), Median (IQR)	26.3 (24.0, 29.6)	25.5 (23.4, 28.9)	26.8 (24.1, 29.7)	26.7 (24.3, 30.0)	<0.001
PD duration (years), Median (IQR)	2.1 (1.2, 3.4)	2.0 (1.2, 3.3)	2.2 (1.2, 3.6)	2.0 (1.1, 3.2)	0.179
PD genetic type, *n* (%)					0.024
Sporadic	1,009 (76.9)	358 (81.9)	332 (76.3)	319 (72.5)	
Non-GBA mutation	199 (15.2)	53 (12.1)	66 (15.2)	80 (18.2)	
GBA mutation	104 (7.9)	26 (5.9)	37 (8.5)	41 (9.3)	
Creatinine (mmol/l), Mean ± SD	82.1 ± 17.8	75.7 ± 13.8	81.4 ± 15.0	89.2 ± 21.1	<0.001
Serum glucose (mmol/l), Mean ± SD	5.6 ± 1.2	5.5 ± 1.2	5.6 ± 1.1	5.6 ± 1.2	0.443
ALT (U/L), Mean ± SD	21.7 ± 12.5	21.2 ± 11.0	22.3 ± 14.7	21.6 ± 11.5	0.449
Serum uric acid (mmol/l), Mean ± SD	305.0 ± 76.2	290.0 ± 71.1	309.4 ± 75.0	315.5 ± 80.0	<0.001
Urea nitrogen (mg/dl), Mean ± SD	16.9 ± 4.6	12.3 ± 2.0	16.5 ± 1.6	21.7 ± 3.5	<0.001
Albumin (g/l), Mean ± SD	44.0 ± 3.5	45.4 ± 3.2	44.0 ± 3.4	42.5 ± 3.3	<0.001
MoCA, Mean ± SD	26.8 ± 2.7	27.2 ± 2.4	26.7 ± 2.8	26.5 ± 2.7	<0.001
MoCA cognitive status, *n* (%)					0.02
Cognitive impairment	341 (26.0)	95 (21.7)	114 (26.2)	132 (30)	
No cognitive impairment	971 (74.0)	342 (78.3)	321 (73.8)	308 (70)	
HVLT-R total recall, Mean ± SD	11.4 ± 2.9	11.6 ± 2.9	11.3 ± 2.9	11.5 ± 3.0	0.212
HVLT-R delayed recall, Mean ± SD	45.9 ± 11.1	46.6 ± 11.5	45.4 ± 10.9	45.6 ± 10.8	0.234
HVLT-R retention, Mean ± SD	45.0 ± 12.1	46.2 ± 12.2	44.4 ± 12.1	44.2 ± 12.0	0.025
HVLT-R discrimination index, Mean ± SD	46.2 ± 12.2	47.4 ± 12.5	45.9 ± 12.2	45.4 ± 11.8	0.044
HVLT-R total recall, Mean ± SD	46.3 ± 11.2	47.2 ± 11.3	45.7 ± 11.2	46.0 ± 11.1	0.098
FAS, Mean ± SD	10.7 ± 3.1	10.9 ± 2.9	10.6 ± 3.1	10.8 ± 3.3	0.539
MSS, Mean ± SD	11.7 ± 3.0	11.9 ± 2.9	11.7 ± 3.0	11.6 ± 3.2	0.262
BNT, Mean ± SD	11.1 ± 2.7	10.9 ± 2.6	11.1 ± 2.8	11.4 ± 2.6	0.198
SDM, Mean ± SD	45.8 ± 10.2	46.8 ± 10.7	45.4 ± 10.2	45.1 ± 9.7	0.032
TMTA, Median (IQR)	0.1 (−0.8, 0.7)	0.1 (−1.0, 0.7)	0.2 (−0.8, 0.8)	0.1 (−0.7, 0.7)	0.594
TMTB, Median (IQR)	−0.1 (−1.5, 0.7)	0.0 (−1.0, 0.6)	−0.3 (−2.2, 0.7)	0.0 (−1.8, 0.8)	0.249
Clock Drawing, Median (IQR)	2.3 (1.0, 2.3)	2.3 (1.0, 2.3)	2.3 (1.3, 2.3)	2.3 (1.0, 2.3)	0.444
LNS, Mean ± SD	11.4 ± 2.9	11.6 ± 2.9	11.3 ± 2.9	11.5 ± 3.0	0.212

N, number; T1: 1.489–3.265; T2: 3.265–4.255; T3: 4.255–10; BMI, body mass index; PD, Parkinson’s disease; race other, includes multi-racial; MoCA, Montreal Cognitive Assessment; MoCA Cognitive status, Cognitive impairment was defined using a MoCA score cutoff of 26; Clock Drawing, Clock Drawing Test *T*-score (age-corrected); HVLT-R Total Recall, Hopkins Verbal Learning Test-Revised (HVLT-R) Immediate/Total Recall *T*-score; HVLT-R Delayed Recall, HVLT-R Delayed Recall *T*-score; HVLT-R Retention, HVLT-R Retention *T*-score; HVLT-R Discrimination Index, HVLT-R Discrimination Index *T*-score; Semantic Fluency (Animal), Semantic Fluency (Animal) *T*-score (age and education-corrected); LNS, Letter-Number Sequencing *T*-score (age-corrected); MSS, Benton Judgment of Line Orientation MOANS Scale Score (age and education-corrected); BNT, Boston Naming Test Scale Score (age and education-corrected); SDM, Symbol-Digit Modalities Test *T*-score (age and education-corrected); FAS, Letter Fluency Test (FAS) *T*-score (Age and Education-Corrected); TMTA, Trail Making Test Part A Reverse *Z*-score (age and education-corrected); TMTB, Trail Making Test Part B Reverse *Z*-score (age and education-corrected). Numbers that do not add up to 100% are attributable to missing data.

### 3.2 Multivariate analysis of BAR and cognitive impairment in PD patients

As shown in Table 2, multivariate linear regression analysis revealed a significant inverse association between BAR (per 1-unit increase) and MoCA scores in PD patients (*β* = −0.21, 95% CI: −0.35 to −0.07, *p* = 0.003) after full covariate adjustment (Model 3). When analyzed by BAR tertiles, patients in the highest tertile (T3: 4.255–10) exhibited a more pronounced reduction in MoCA scores (β = −0.44, 95% CI: −0.81 to −0.07, *p* = 0.019) compared to the lowest tertile (T1: 1.489–3.265), while the middle tertile (T2: 3.265–4.255) showed a marginal trend (*β* = −0.34, 95% CI: −0.68 to 0.01, *p* = 0.06).

**Table 2:** Linear regression analysis of BAR and MoCA score in PD patients.

Variable	*n* total	Model 1		Model 2		Model 3	
		*β* (95%CI)	*p*-value	*β* (95%CI)	*p*-value	*β* (95%CI)	*p*-value
BAR	1,312	−0.28 (−0.41 ~ −0.16)	<0.001	−0.14 (−0.27 ~ −0.02)	0.028	−0.21 (−0.35 ~ −0.07)	0.003
Tertiles							
T1	437	0(Ref)		0(Ref)		0(Ref)	
T2	435	−0.50 (−0.85 ~ −0.15)	0.005	−0.30 (−0.64 ~ 0.05)	0.095	−0.34 (−0.68 ~ 0.01)	0.06
T3	440	−0.69 (−1.04 ~ −0.34)	<0.001	−0.34 (−0.7 ~ 0.01)	0.061	−0.44 (−0.81 ~ −0.07)	0.019
*p* for trend	1,312		<0.001		0.061		0.019

N, number; T1: 1.489–3.265; T2: 3.265–4.255; T3: 4.255–10; BAR, Blood Urea Nitrogen to Serum Albumin Ratio; PD, Parkinson’s disease; BMI, body mass index; ALT, alanine aminotransferase. Model 1: unadjusted. Model 2: Adjusted factors include age, sex, race, BMI. Model 3: Adjusted age, sex, race, BMI, serum glucose, creatinine, ALT, serum uric acid.

**Figure 2:**
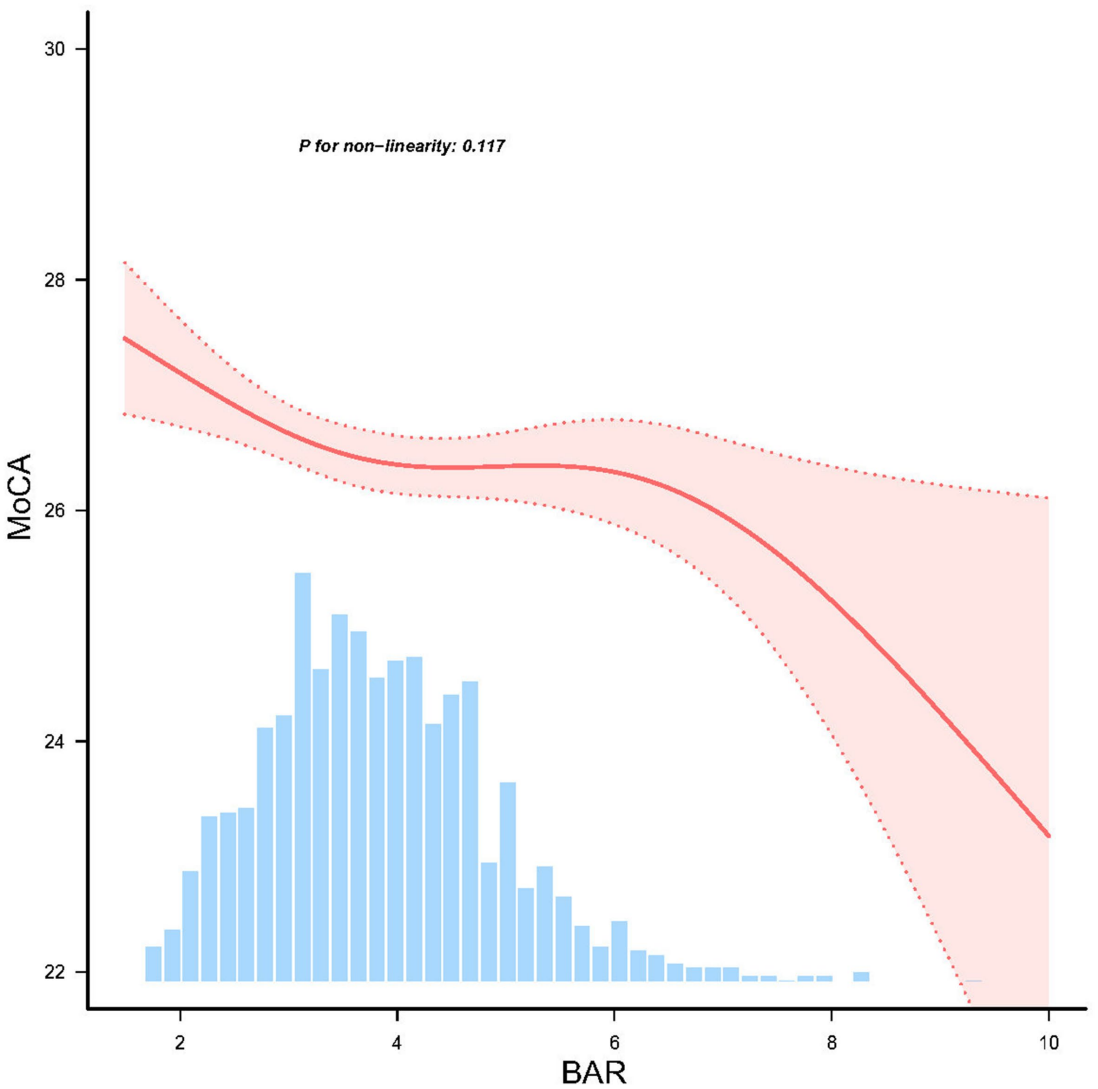
Linear dose response relationship between BAR and cognitive impairment in PD patients, adjusted for age, sex, race, and body mass index (BMI), ALT, creatinine, serum uric acid, and serum glucose. The red line and red area represent the estimated values and their corresponding 95% confidence intervals, respectively.

RCS analysis confirmed a linear dose–response relationship between BAR and MoCA scores (*P* for nonlinearity = 0.117) (Figure 2), suggesting no significant threshold or saturation effects.

We categorized PD patients into two groups based on the MoCA score, using a cutoff of 26 points: those with cognitive impairment (MoCA score < 26) and those without cognitive impairment (MoCA score ≥ 26). Multivariate logistic regression analysis revealed that the BAR was significantly associated with cognitive impairment, with an odds ratio (OR) of 1.15 (95% CI: 1.02–1.30, *p* = 0.027) (Table 3).

**Table 3:** Logistic regression analysis of BAR and MoCA-determined cognitive impairment.

Variable	*n* total	Model 1		Model 2		Model 3	
		OR (95%CI)	*p*-value	OR (95%CI)	*p*-value	OR (95%CI)	*p*-value
Cognitive impairment	1,312	1.20 (1.08 ~ 1.33)	0.001	1.11 (0.99 ~ 1.24)	0.077	1.15 (1.02 ~ 1.30)	0.027

Cognitive impairment was defined using a MoCA score cutoff of 26, with scores below 26 indicating the presence of cognitive impairment. n, number; BAR, blood urea nitrogen to serum albumin ratio; PD, Parkinson’s disease; BMI, body mass index; ALT, alanine aminotransferase. Model 1: unadjusted. Model 2: Adjusted factors include age, sex, race, BMI. Model 3: Adjusted age, sex, race, BMI, serum glucose, creatinine, ALT, serum uric acid.

To further elucidate the relationship between BAR and cognitive impairment, we conducted linear regression analyses with other cognitive assessment metrics (Tables 4–6). The results indicated that BAR was negatively associated with the Benton Judgment of Line Orientation MOANS Scale Score (*β* = −0.25, 95% CI: −0.40 to −0.10, *p* = 0.001). BAR exhibited a negative association with the Symbol-Digit Modalities Test T-score (*β* = −0.97, 95% CI: −1.49 to −0.44, *p* < 0.001). Similarly, BAR demonstrated a positive association with the Trail Making Test Part A Reverse *Z*-score (*β* = 0.08, 95% CI: 0.02–0.15, *p* = 0.011).

**Table 4:** Linear regression analysis of BAR and other cognitive test results.

Variable	*n* total	Model 1		Model 2		Model 3	
		*β* (95%CI)	*p-*value	*β* (95%CI)	*p-*value	β (95%CI)	*p-*value
HVLT-R total recall	1,312	−0.13 (−0.65 ~ 0.40)	0.639	−0.18 (−0.70 ~ 0.33)	0.481	−0.13 (−0.69 ~ 0.42)	0.642
HVLT-R delayed recall	1,312	−0.42 (−0.99 ~ 0.16)	0.157	−0.27 (−0.84 ~ 0.30)	0.359	−0.27 (−0.89 ~ 0.34)	0.385
HVLT-R retention	1,312	−0.47 (−1.05 ~ 0.11)	0.113	−0.29 (−0.89 ~ 0.30)	0.334	−0.45 (−1.09 ~ 0.20)	0.176
HVLT-R discrimination index	1,312	−0.4 (−0.93 ~ 0.13)	0.142	−0.04 (−0.57 ~ 0.50)	0.897	0.06 (−0.52 ~ 0.64)	0.832

n, number; BAR, blood urea nitrogen to serum albumin ratio; PD, Parkinson’s disease; BMI, body mass index; ALT, alanine aminotransferase; HVLT-R Total Recall, Hopkins Verbal Learning Test-Revised (HVLT-R) Immediate/Total Recall *T*-score; HVLT-R Delayed Recall, HVLT-R Delayed Recall *T*-score; HVLT-R Retention, HVLT-R Retention *T*-score; HVLT-R Discrimination Index, HVLT-R Discrimination Index *T*-score. Model 1: unadjusted. Model 2: Adjusted factors include age, sex, education years, race, BMI. Model 3: Adjusted age, sex, education years, race, BMI, serum glucose, creatinine, ALT, serum uric acid.

**Table 5:** Linear regression analysis of BAR and other cognitive test results adjusted for age and education.

Variable	*n* total	Model 1		Model 2		Model 3	
		*β* (95%CI)	*p-*value	*β* (95%CI)	*p-*value	*β* (95%CI)	*p-*value
FAS	1,312	−0.07 (−0.21 ~ 0.07)	0.321	−0.06 (−0.21 ~ 0.08)	0.378	−0.06 (−0.22 ~ 0.09)	0.422
MSS	1,312	−0.17 (−0.31 ~ −0.03)	0.021	−0.26 (−0.40 ~ −0.12)	<0.001	−0.25 (−0.40 ~ −0.10)	0.001
BNT	1,312	−0.03 (−0.20 ~ 0.14)	0.744	−0.04 (−0.21 ~ 0.13)	0.66	−0.03 (−0.22 ~ 0.16)	0.729
SDM	1,312	−0.71 (−1.20 ~ −0.23)	0.004	−0.58 (−1.06 ~ −0.10)	0.019	−0.97 (−1.49 ~ −0.44)	<0.001
Semantic fluency (Animal)	1,312	−0.31 (−0.81 ~ 0.19)	0.224	−0.29 (−0.79 ~ 0.21)	0.26	−0.26 (−0.81 ~ 0.29)	0.349
TMTA	1,312	0.11 (0.05 ~ 0.17)	<0.001	0.11 (0.05 ~ 0.17)	<0.001	0.08 (0.02 ~ 0.15)	0.011
TMTB	1,312	0.01 (−0.06 ~ 0.09)	0.701	0.02 (−0.05 ~ 0.10)	0.515	0 (−0.08 ~ 0.08)	0.962

n, number; BAR, blood urea nitrogen to serum albumin ratio; PD, Parkinson’s disease; BMI, body mass index; ALT, alanine aminotransferase; Semantic Fluency (Animal), Semantic Fluency (Animal) *T*-score (age and education-corrected); MSS, Benton Judgment of Line Orientation MOANS Scale Score (age and education-corrected); BNT, Boston Naming Test Scale Score (age and education-corrected); SDM, Symbol-Digit Modalities Test *T*-score (age and education-corrected); FAS, Letter Fluency Test (FAS) *T*-score (Age and Education-Corrected); TMTA, Trail Making Test Part A Reverse Z-score (age and education-corrected); TMTB, Trail Making Test Part B Reverse *Z*-score (age and education-corrected). Model 1: unadjusted. Model 2: Adjusted factors include sex, race, BMI. Model 3: Adjusted sex, race, BMI, serum glucose, creatinine, ALT, serum uric acid.

**Table 6:** Linear regression analysis of BAR and other cognitive test results adjusted for age.

Variable	*n* total	Model 1		Model 2		Model 3	
		*β* (95%CI)	*p-*value	*β* (95%CI)	*p-*value	*β* (95%CI)	*p*-value
Clock drawing	1,312	−0.62 (−1.33 ~ 0.10)	0.091	−0.49 (−1.21 ~ 0.22)	0.174	−0.32 (−1.10 ~ 0.45)	0.413
LNS	1,312	−0.02 (−0.16 ~ 0.12)	0.758	−0.05 (−0.18 ~ 0.09)	0.492	−0.11 (−0.25 ~ 0.04)	0.157

n, number; BAR, blood urea nitrogen to serum albumin ratio; PD, Parkinson’s disease; BMI, body mass index; ALT, alanine aminotransferase; Clock Drawing, Clock Drawing Test *T*-score (age-corrected); LNS, Letter-Number Sequencing *T*-score (age-corrected). Model 1: unadjusted. Model 2: Adjusted factors include education years, sex, race, BMI. Model 3: Adjusted education years, sex, race, BMI, serum glucose, creatinine, ALT, serum uric acid.

### 3.3 Subgroup and sensitivity analyses

**Figure 3:**
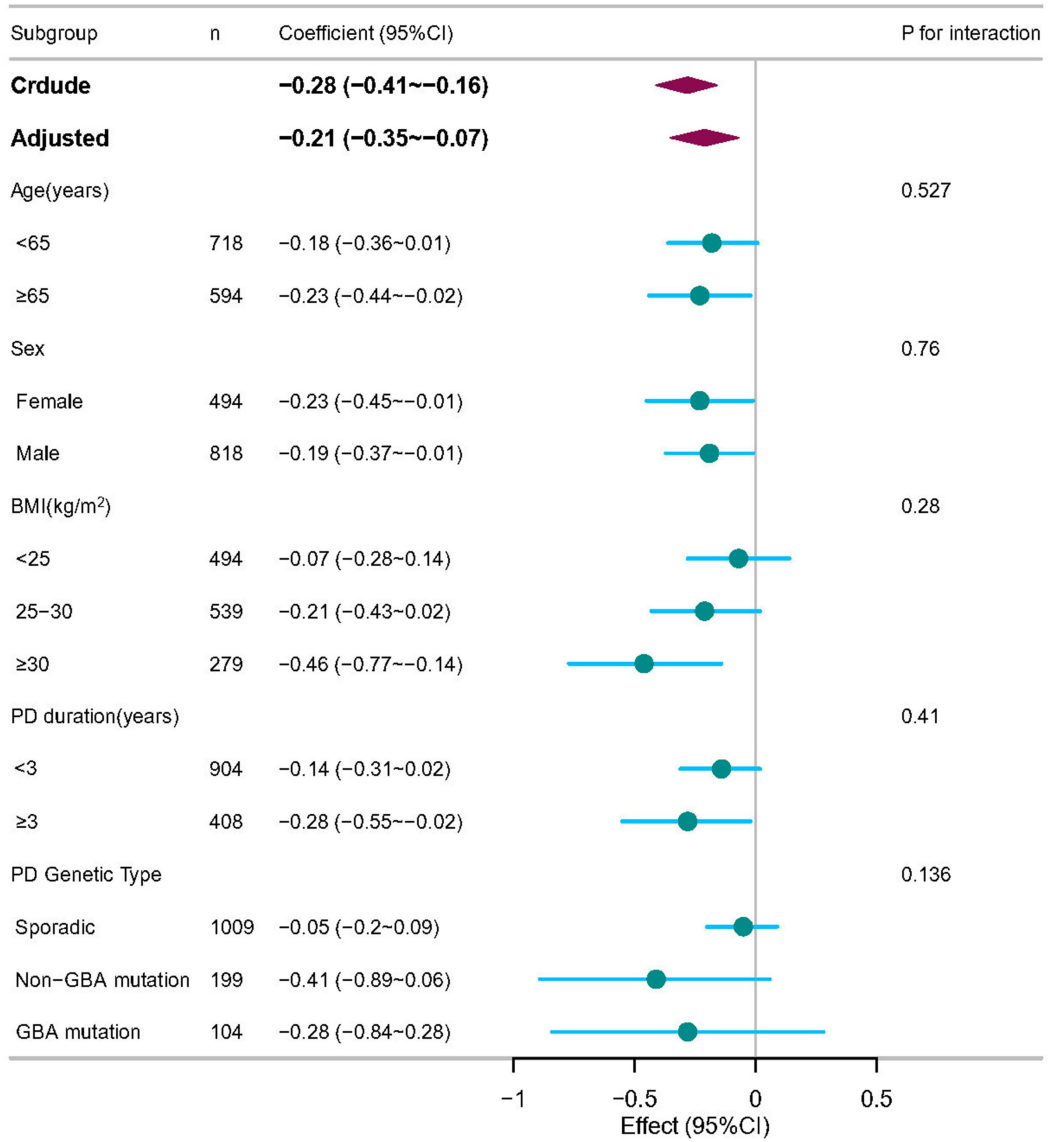
Subgroup analyses of the associations between BAR and cognitive impairment in PD patients, adjusted for age, sex, race, and body mass index (BMI), ALT, creatinine, serum uric acid, and serum glucose. In each case, the model was not adjusted for the stratification variable.

To assess potential effect modification, we conducted stratified analyses by age, sex, BMI, PD duration, and PD genetic subtype (Figure 3). No significant interactions were observed between BAR and these variables in relation to MoCA scores (*P* for interaction >0.05 for all), suggesting that the association was consistent across subgroups.

To evaluate the robustness of our findings, we performed sensitivity analyses by excluding participants with missing covariate data. The inverse association between BAR and MoCA scores remained significant (*β* = −0.22, 95% CI: −0.36 to −0.08), further supporting the primary results (Table 7). Similarly, when participants with renal insufficiency were excluded, the inverse association was still significant (*β* = −0.21, 95% CI: −0.35 to −0.07) (Table 8).

**Table 7:** Linear Regression Analysis of BAR and MoCA score in PD patients without missing data.

Variable	*n* total	Model 1		Model 2		Model 3	
		*β* (95%CI)	*p-*value	*Β* (95%CI)	*p*-value	*β* (95%CI)	*p-*value
BAR	1,286	−0.28 (−0.41 ~ −0.16)	<0.001	−0.15 (−0.28 ~ −0.02)	0.028	−0.22 (−0.36 ~ −0.08)	0.002
Tertiles							
T1	424	0(Ref)		0(Ref)		0(Ref)	
T2	429	−0.50 (−0.85 ~ −0.15)	0.005	−0.28 (−0.62 ~ 0.07)	0.123	−0.31 (−0.66 ~ 0.04)	0.081
T3	433	−0.69 (−1.04 ~ −0.34)	<0.001	−0.36 (−0.72 ~ 0)	0.052	−0.46 (−0.83 ~ −0.08)	0.016
*p* for trend	1,286		<0.001		0.052		0.016

n, number; T1: 1.489–3.265; T2: 3.265–4.255; T3: 4.255–10; BAR, blood urea nitrogen to serum albumin ratio; PD, Parkinson’s disease; BMI, body mass index; ALT, alanine aminotransferase. Model 1: unadjusted. Model 2: Adjusted factors include age, sex, race, BMI. Model 3: Adjusted age, sex, race, BMI, serum glucose, creatinine, ALT, serum uric acid.

**Table 8:** Linear regression analysis of BAR and MoCA score in PD patients without patients with renal impairment.

Variable	*n* total	Model 1		Model 2		Model 3	
		*β* (95%CI)	*p-*value	*Β* (95%CI)	*p-*value	*β* (95%CI)	*p* value
BAR	1,286	−0.30 (−0.43 ~ −0.17)	<0.001	−0.16 (−0.30 ~ −0.03)	0.017	−0.21 (−0.35 ~ −0.07)	0.004
Tertiles							
T1	435	0(Ref)		0(Ref)		0(Ref)	
T2	432	−0.50 (−0.85 ~ −0.14)	0.006	−0.3 (−0.65 ~ 0.05)	0.097	−0.33 (−0.68 ~ 0.02)	0.066
T3	419	−0.73 (−1.09 ~ −0.38)	<0.001	−0.39 (−0.75 ~ −0.02)	0.038	−0.46 (−0.84 ~ −0.09)	0.016
*p* for trend	1,286		<0.001		0.037		0.015

n, number; T1: 1.489–3.265; T2: 3.265–4.255; T3: 4.255–10; BAR, blood urea nitrogen to serum albumin ratio; PD, Parkinson’s disease; BMI, body mass index; ALT, alanine aminotransferase. Model 1: unadjusted. Model 2: Adjusted factors include age, sex, race, BMI. Model 3: Adjusted age, sex, race, BMI, serum glucose, creatinine, ALT, serum uric acid.

### 3.4 Longitudinal cognitive trajectories and their association with baseline BAR

**Figure 4:**
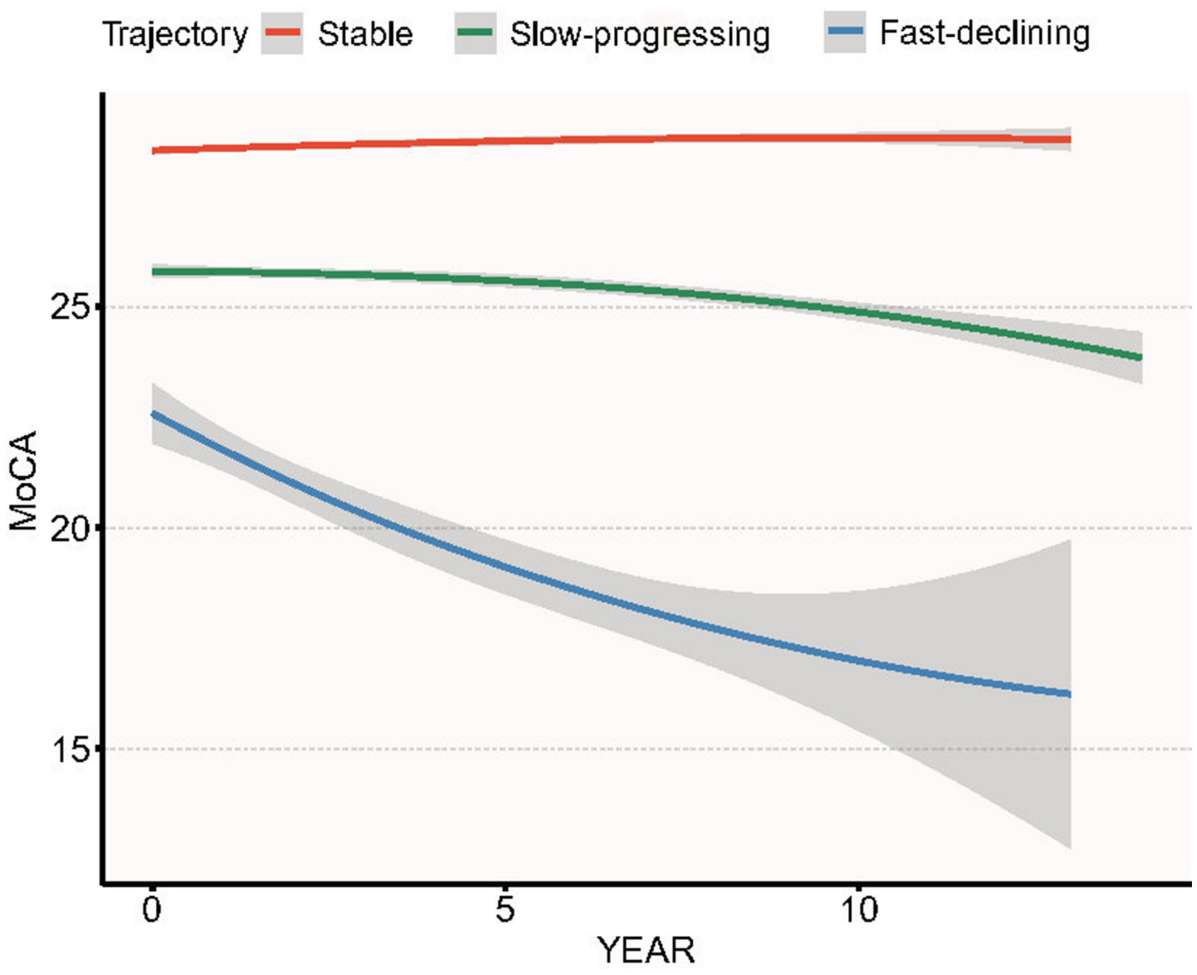
Group-based trajectories of MoCA score over 14 years of follow-up in PPMI cohort.

Over 14 years of follow-up we identified three distinct MoCA trajectories (Figure 4): Stable-MoCA (46%, *n* = 607) remained virtually flat; Slow-progressing MoCA (44%, *n* = 574) declined gradually; and Fast-declining MoCA (10%, *n* = 131) showed an accelerated drop. Higher baseline BAR was associated with a shift toward worse cognitive trajectories after adjustment for age, sex, race, BMI, serum glucose, creatinine, ALT, serum uric acid (Table 9). Per 1-SD increase in baseline BAR, the odds of belonging to the Slow-progressing MoCA trajectory versus the Stable-MoCA trajectory were 1.32 (95% CI 1.17–1.49, *p* < 0.001), and the odds of belonging to the Fast-declining MoCA trajectory versus the Stable-MoCA trajectory were 1.47 (95% CI 1.22–1.78, *p* < 0.001).

**Table 9:** Multinomial logistic regression of baseline BAR (per 1-SD increase) and 14-year MoCA trajectory membership in early Parkinson’s disease.

Variable	*n* total	Model 1		Model 2		Model 3	
		OR (95%CI)	*p-*value	OR (95%CI)	*p-*value	OR (95%CI)	*p-*value
Longitudinal MoCA trajectory	1,312						
Stable	607	0(Ref)		0(Ref)		0(Ref)	
Slow-progressing	574	1.41 (1.27 ~ 1.58)	<0.001	1.3 (1.16 ~ 1.45)	<0.001	1.32 (1.17 ~ 1.49)	<0.001
Fast-declining	131	1.51 (1.28 ~ 1.78)	<0.001	1.32 (1.11 ~ 1.57)	0.002	1.47 (1.22 ~ 1.78)	<0.001

Model 1: unadjusted. Model 2: Adjusted factors include age, sex, race, BMI. Model 3: Adjusted age, SEX, race, BMI, serum glucose, creatinine, ALT, serum uric acid.

## 4 Discussion

This study established a significant and independent link between higher BAR levels and cognitive impairment in PD patients. Specifically, higher BAR levels correlated negatively with MoCA scores (*p* = 0.003), suggesting that elevated BAR may indicate cognitive decline in PD. Results were consistent across clinical subgroups and in sensitivity analyses. Specifically, a higher BAR was associated with cognitive impairment as defined by a MoCA score cutoff of 26, indicating that elevated BAR levels may be indicative of cognitive decline in PD patients. Further analysis revealed that BAR was negatively correlated with the Benton Judgment of Line Orientation MOANS Scale Score and the Symbol-Digit Modalities Test *T*-score. These findings suggest that higher BAR levels are associated with poorer performance in visuospatial abilities and processing speed, which are critical cognitive domains often affected in PD. Conversely, BAR was positively correlated with the Trail Making Test Part A Reverse *Z*-score, indicating that higher BAR levels may be linked to slower visual search speed and motor speed, further supporting the notion that BAR reflects cognitive and motor dysfunction in PD. Our results highlight the potential utility of BAR as a biomarker for cognitive impairment in PD, with significant associations observed across multiple cognitive domains.

Studies directly examining the association between the BAR and cognitive impairment in PD patients are currently nonexistent. Elevated BUN levels correlate with cognitive impairment in animal models (*p* < 0.05) and the elderly with diabetes, while paradoxically lower values are observed in Alzheimer’s disease (AD)-related dementia (5.25 ± 1.23 mmoL/L) compared to AD-related mild cognitive impairment (6.08 ± 1.15 mmoL/L) (*p* = 0.003) (26–28). Previous studies on maintenance hemodialysis patients have shown that those with cognitive impairment have significantly higher BUN levels (30.69 ± 6.17 mmoL/L) compared to those without cognitive impairment (21.19 ± 7.39 mmoL/L, *p* < 0.05) (29). Additionally, research utilizing the NHANES database has identified BUN as a significant predictor of 5-year survival rates in individuals with cognitive impairment (30). A single-center study from Japan also found that, compared to patients in the low tertile of albumin levels, those in the middle tertile had higher Mini-Mental State Examination (MMSE) scores after adjusting for confounding factors (31). Data from England also indicate that low serum albumin is independently associated with an increased likelihood of cognitive dysfunction in the elderly population (32). Recent research has also identified a connection between the BAR and cerebral small vessel disease (cSVD), a condition often associated with cognitive impairment (18) found that the BAR is associated with all types of cSVD in health check-up participants. Another study demonstrated that both AD pathology (Aβ) and cSVD are linked to impaired choroid plexus cerebrospinal fluid drainage, which in turn is associated with cognitive impairment (33). These findings suggest a potential pathway through which the BAR may influence cognitive function. Despite the lack of prior studies directly examining the correlation between the BAR and cognitive impairment in PD patients, the existing data align with our findings, supporting the notion that a higher BAR is associated with cognitive impairment.

BUN, an end-product of protein metabolism synthesized in the liver and excreted by the kidneys, has been shown to have associations across neurocognitive disorders. BUN levels may reflect poor nutritional status and metabolic imbalance when renal function is normal and there is no excessive protein intake. BUN is increasingly recognized as a key marker of metabolic disturbances, including insulin resistance (34), oxidative stress (35), and inflammation (36), which can lead to endothelial dysfunction and vascular damage. Elevated BUN levels can cause arterial endothelial dysfunction (37), stimulate pro-atherosclerotic pathways, and promote endothelial progenitor cell senescence (38). Oxidative stress and inflammation are associated with neurodegeneration, including cognitive impairment (39). Atherosclerosis, which can be exacerbated by elevated BUN levels, is a significant factor in cognitive impairment among the elderly (40, 41).

Albumin, the most abundant plasma protein and a major component of cerebrospinal fluid, has multifunctional properties, including antioxidant function, immune regulation, anti-inflammatory activity, and endothelial stabilization (42). Albumin also has neuroprotective effects, partly due to its ability to modulate intracellular signaling and antioxidant properties in neurons or glial cells (43). In AD, albumin can suppress amyloid formation and block further accumulation of peptide amyloid beta (Aβ) protein; serum albumin levels are inversely associated with Aβ deposition and Aβ positivity (44). In PD, *α*-synuclein is a key factor that may further trigger oxidative stress and neuroinflammation, both of which play important roles in the pathogenesis of PD (45). Human serum albumin can hinder the fibrillation process of α-synuclein, mitigate membrane damage caused by α-synuclein, and significantly reduce α-synuclein aggregation at concentrations found in human serum (46).

Beyond metabolic and vascular pathways, it remains possible that BAR-associated cognitive vulnerability is modulated by Parkinson’s-specific genetics. Recent translational work indicates that GBA mutations impair lysosomal α-synuclein clearance, while oxidative-stress-related polymorphisms in WWOX and MAF heighten cortical susceptibility to neurodegeneration (47–50). These findings raise the hypothesis that metabolic stress reflected by elevated BAR could interact with genetically determined defects in protein handling and antioxidant defense, thereby accelerating cognitive decline in PD. Our PPMI sample is enriched for both sporadic and GBA-positive early-stage patients, yet the present analysis was not powered to test gene–metabolite interactions. Future studies coupling targeted genotyping (GBA, WWOX, MAF, etc.) with longitudinal metabolic profiling will be required to determine whether BAR adds predictive value specifically in genetically defined high-risk subgroups.

This study offers valuable insights into the relationship between the BAR and cognitive impairment in PD patients. The BAR, which integrates the clinical value of BUN and albumin, provides a comprehensive assessment that may be more useful than either BUN or serum albumin alone. It is simpler to calculate, less subjective, and more convenient for clinical use. Our investigation employed multivariate linear regression models to explore the association between BAR and cognitive impairment in PD patients, effectively controlling for potential confounders and reducing bias. Multivariate logistic regression analysis further confirmed that BAR is associated with cognitive impairment as defined by MoCA scores, solidifying the link between BAR and cognitive status. Our analysis extended to specific cognitive domains, demonstrating that BAR correlates with the Benton Judgment of Line Orientation MOANS Scale Score and the Symbol-Digit Modalities Test T-score, indicating its relevance across multiple facets of cognitive function. To gain a comprehensive understanding of the relationship between BAR and cognitive impairment in PD patients, we performed smooth curve fitting to illustrate the linear association. Additionally, the robustness of our findings was verified through stratified subgroup and sensitivity analyses, which examined the relationship across different populations.

Despite the valuable insights our study provides into the relationship between BAR and cognitive impairment in PD, several limitations must be acknowledged. The observational nature of our research restricts its direct comparability to the gold standard of randomized controlled trials. Additionally, the cross-sectional design limits our ability to fully understand the causal mechanisms underlying the observed associations. Our study identifies a correlation rather than a causal link between BAR and cognitive impairment in PD, emphasizing the need for future prospective cohort studies to validate our findings. Despite constructing regression models and conducting stratified and sensitivity analyses, we cannot entirely rule out residual confounding effects from unmeasured or unknown factors. The retrospective nature of the study may also introduce biases inherent to this design. Although longitudinal trajectory modelling of the PPMI follow-up data corroborated the cross-sectional association between BAR and cognitive decline, the mild trajectories observed here probably reflect selection and attrition biases inherent to the PPMI cohort. Future research should aim to address these limitations through more robust study designs, including longitudinal data, to further elucidate the relationship between BAR and the progression of cognitive impairment in PD. Longitudinal studies are essential to establish causality and to better understand the dynamic relationship between BAR and cognitive decline over time. Additionally, mechanistic investigations are imperative to substantiate our results and to explore the biological pathways through which BAR may influence cognitive function in PD.

## 5 Conclusion

This study highlights the significant impact of BAR levels on the risk of cognitive impairment in PD, independent of other confounding variables. Our findings demonstrate a clear linear relationship between BAR and cognitive impairment in PD, suggesting that higher BAR levels are associated with a greater risk of cognitive decline. These results are of substantial interest and may have crucial implications for understanding the pathogenesis of cognitive impairment in PD and the development of disease-modifying therapies. Future research should aim to address the limitations of our study and to provide a deeper understanding of the role of BAR in the progression of cognitive impairment in PD.

Data will be accessible upon request to facilitate additional research and scrutiny.

## Data Availability

Publicly available datasets were analyzed in this study. This data can be found here: http://www.ppmi-info.org.

## Footnotes

1
http://www.ppmi-info.org

2
http://www.R-project.org

3
http://www.clinicalscientists.cn/freestatistics
